# MicroRNA miR-274-5p Suppresses *Found-in-Neurons* Associated with Melanotic Mass Formation and Developmental Growth in *Drosophila*

**DOI:** 10.3390/insects14080709

**Published:** 2023-08-14

**Authors:** Hee Kyung Kim, Chae Jeong Kim, Daegyu Jang, Do-Hwan Lim

**Affiliations:** School of Systems Biomedical Science, Soongsil University, Seoul 06978, Republic of Korea; gmlruddl200@naver.com (H.K.K.); jinrourin313@naver.com (C.J.K.); sjj609349@gmail.com (D.J.)

**Keywords:** *Drosophila*, miR-274, melanotic mass, growth, JNK signaling, JAK/STAT signaling, *found-in-neurons*

## Abstract

**Simple Summary:**

In animals, including humans and flies, blood cells play crucial roles in various biological processes, such as immune response and normal development. The abnormal regulation of hematopoiesis in flies results in the formation of melanotic masses, which are also known as melanotic tumors. However, the detailed mechanisms underlying this process are not fully understood. In this study, we found that the upregulation of miR-274-5p, a small non-coding RNA, activates the JNK and JAK/STAT signaling pathways by suppressing the expression of *found-in-neurons* (*fne*) encoding an RNA-binding protein in flies. This regulation controls the formation of melanotic masses and developmental growth. Overall, our findings provide valuable insights into melanotic mass formation and developmental growth as well as the regulatory network of the JNK and JAK/STAT signaling pathways.

**Abstract:**

The hematopoietic system plays a crucial role in immune defense response and normal development, and it is regulated by various factors from other tissues. The dysregulation of hematopoiesis is associated with melanotic mass formation; however, the molecular mechanisms underlying this process are poorly understood. Here, we observed that the overexpression of *miR-274* in the fat body resulted in the formation of melanotic masses. Moreover, abnormal activation of the JNK and JAK/STAT signaling pathways was linked to these consequences. In addition to this defect, *miR-274* overexpression in the larval fat body decreased the total tissue size, leading to a reduction in body weight. miR-274-5p was found to directly suppress the expression of *found-in-neurons* (*fne*), which encodes an RNA-binding protein. Similar to the effects of *miR-274* overexpression, *fne* depletion led to melanotic mass formation and growth reduction. Collectively, miR-274 plays a regulatory role in the *fne*–JNK signaling axis in melanotic mass formation and growth control.

## 1. Introduction

Blood cells play important roles in the immune response against invading pathogens and in the normal development of metazoan [1,2]. In *Drosophila*, hematopoiesis occurs in two different waves: in the head mesoderm of early embryos and the lymph glands of larvae [3,4,5]. During this process, *Drosophila* prohemocytes terminally differentiate into three types of blood cells: plasmatocytes (phagocytosis), crystal cells (melanization), and lamellocytes (encapsulation) [2,5]. Phagocytic plasmatocytes engulf apoptotic bodies and pathogens, such as bacteria and fungi, as the predominant hemocytes [6,7]. Lamellocytes, which are rare in healthy conditions, can massively differentiate after infection and form a capsule around foreign pathogens [2,8]. Melanization is facilitated by crystal cells that secrete phenol oxidase [8]. This hemocyte-mediated cellular immune response is involved in the formation of larval melanotic masses.

In *Drosophila*, melanotic mass formation, a conspicuous cellular response, can be induced via abnormal immune responses, such as lymph gland overgrowth and the massive differentiation of lamellocytes [2,9]. The formation of melanotic masses is closely linked to several signaling pathways, including the Toll, Janus kinase/signal transducer and activator of transcription (JAK/STAT), *Drosophila* immune deficiency (IMD)/Relish, and Ras/mitogen-activated protein kinase (Ras/MAPK) pathways [8]. The JAK/STAT signaling pathway is associated with innate immunity and hematopoiesis. According to a model of the JAK/STAT signaling pathway in *Drosophila*, binding of the cytokine, Unpaired (Upd), to the Domeless (Dome) receptor induces receptor dimerization and activation of the JAK Hopscotch (Hop in *Drosophila*). Activated Hops phosphorylate STAT proteins, allowing them to form dimers and translocate into the nucleus to regulate the expression of target genes, such as the *Suppressor of cytokine signaling at 36E* (*Scocs36E*) [10]. Loss of JAK/STAT signaling leads to impaired encapsulation, whereas aberrant activation of the JAK/STAT pathway results in premature lamellocyte differentiation and melanotic mass formation [10]. The activation of c-Jun N-terminal Kinase (JNK) signaling can affect the JAK/STAT signaling pathway by inducing the expression of *Upds*, which is a ligand of the JAK/STAT pathway [11]. These circulating Upds can also be released from other larval tissues, including the fat body and injured tissues, triggering the activation of the JAK/STAT signaling pathway in the target tissues [10,11]. This occurs through an amplification loop that mutually activates the JAK/STAT signaling in each tissue [10,11]. Furthermore, these processes can affect hemocytes [11]. However, the regulation of hematopoiesis associated with melanotic mass formation is still poorly understood.

*Drosophila* undergoes growth until a distinct stage, known as the larval-to-pupal transition, which is triggered by the ecdysone steroid hormone. The final body size of *Drosophila* is influenced by the growth rate during this stage and the duration of growth [12]. Under these conditions, the size and number of cells composing individual tissues ultimately determine the final size [12]. These growth conditions are intricately regulated by complex networks of internal cues, such as hormones and signaling pathways, as well as external factors like nutrition. For instance, the downregulation of *slimfast* (*slif*), which encodes an amino acid transporter, leads to a growth defect through the target of rapamycin (TOR) signaling pathway [13]. However, the complete regulatory networks controlling growth remain incompletely understood.

MicroRNAs (miRNAs) are small non-coding RNAs (~21 nucleotides in length) that post-transcriptionally suppress gene expression by inducing RNA degradation and/or translational repression [14]. After transcription from the genome, primary miRNAs are cleaved into precursor miRNAs (pre-miRNAs) by the Drosha protein [15]. Subsequently, the pre-miRNAs are processed into miRNA duplexes by the Dicer protein [16]. Depending on whether it is derived from the 5′ or 3′ arm of the stem region in the pre-miRNA, each strand of the miRNA duplex is presented with a −5p or −3p suffix, respectively [17]. One strand of the miRNA duplexes is incorporated into the RNA-induced silencing complex containing the Argonaute protein, regulating the expression of target mRNAs by binding to their 3′-untranslated regions (3′-UTR) [18]. Using high-throughput sequencing, a large number of miRNAs have been identified across species, including humans, mice, and flies. According to the miRbase, 469 mature miRNAs have been identified in *Drosophila melanogaster* [19]. Individual miRNAs have been estimated to target approximately 200 mRNA transcripts on average [20]. Through these complicated regulatory integrations, miRNAs are involved in various biological processes including development, growth, metabolism, and cell death [20]. In particular, as an miRNA linked to the JAK/STAT signaling pathway, *Drosophila* miR-279 is involved in ovarian cell fate and circadian behavior by regulating *stat92E* and *upd*, respectively [21,22]. Additionally, miR-306 and miR-79 enhance the activation of JNK signaling by suppressing RNF146, which is an E3 ubiquitin ligase [23]. However, researchers have revealed the roles of only some miRNAs in the signaling pathways controlling various biological processes, and the biological functions of most miRNAs still need to be explored. The *found-in-neurons* (*fne*) gene encodes an RNA-binding protein as one of the three paralogs (Rbp9, Fne, and Elav) of the ELAV gene family, and it is primarily expressed in neuronal tissues in *Drosophila* [24]. According to previous reports, *fne* is associated with several biological processes, such as mushroom body development, male courtship performance, and synaptic plasticity [24,25]. In addition, similar to other family proteins, Fne broadly induces 3′-UTR extension in neuronal cells by blocking the use of the proximal polyadenylation site [26]. The cytoplasmic protein, Fne, undergoes a switch of cellular localization toward the nucleus due to the inclusion of a microexon encoding a nuclear localization signal under Elav-nonfunctional conditions [26]. However, functional studies on Fne have focused on its role in primarily expressed neuronal cells. As a result, other biological roles of *fne* in non-neuronal cells remain unknown.

In *Drosophila*, glia-derived miR-274 is known to play a role in regulating the growth of synaptic boutons and tracheal branches through the targeting of *Sprouty* [27]. Furthermore, miR-274 is known to be upregulated in *Drosophila* under pathogen bacteria, *Micrococcus luteus* infection conditions [28]. However, the biological functions of miR-274 in other contexts and under these upregulated conditions remain unclear. In the present study, we investigated the biological role of *Drosophila* miR-274 in larval fat bodies in terms of melanotic mass formation and developmental growth. We found that miR-274 overexpression results in the activation of the JNK and JAK/STAT signaling pathways, which are closely associated with the observed phenotypic consequences. Furthermore, we revealed that this regulation of miR-274 was mediated by the RNA-binding protein Fne, which is a biologically relevant target of miR-274. Overall, our findings suggest that miR-274 plays a crucial role in regulating the *fne*–JNK signaling axis under its upregulated conditions, which in turn affects melanotic mass formation and developmental growth.

## 2. Materials and Methods

### 2.1. Drosophila Strains

All flies were grown at 25 °C on standard cornmeal/agar medium under noncrowded conditions. Transgenic overexpression studies were performed using the *GAL4/UAS* system. The following fly lines from the Bloomington *Drosophila* Stock Center were used: *w^1118^* (BL5905), *Cg-GAL4* (BL7011), *ppl-GAL4* (BL58768), *UAS-LUC-miR-274* (BL41172), and *UAS-fne-RNAi^TRiP^* (BL28784).

### 2.2. Analysis of Melanotic Mass

All larvae and flies were maintained on standard cornmeal/agar media. Melanotic masses were analyzed in wandering third-instar larvae and adult flies as previously described [29]. For quantitative analysis, the percentage of wandering third-instar larvae with melanotic masses was determined using three vials per genotype. Representative images of wandering third-instar larvae or adult flies were captured using a stereomicroscope (Olympus, Shinjuku-ku, Tokyo, Japan).

### 2.3. Determination of the Eclosion Rate

After the eggs were laid on standard cornmeal/agar media at 25 °C, three vials containing eggs from each genotype were transferred to a 29 °C incubator. More than 130 pupae were analyzed for each genotype at 15 d AEL, and the rate of empty puparia in each vial was calculated to determine the eclosion rate.

### 2.4. RNA Isolation and Determination of RNA Transcript Level

Total RNA was purified from S2 cells, larval fat bodies, and adult heads using the TRI Reagent (Molecular Research Center, Cincinnati, OH, USA), according to the manufacturer’s instructions. TRI Reagent BD (Molecular Research Center) was used for RNA isolation from larval hemocytes. The following protocol was used for each sample: S2 cells were harvested via centrifugation for 5 min at 350× *g*. Fat bodies were dissected in cold phosphate-buffered saline (PBS) from wandering third-instar larvae in cold PBS. To collect hemolymph-containing hemocytes, we used a previously described protocol with some modifications [30]. Briefly, after washing the larvae with distilled water, individual larvae were transferred to cold PBS, and the cuticle was gently torn away to allow the hemolymph to bleed out. Hemolymph was then collected in a new tube.

miRNA quantification was performed using a PCR-based method, as previously described [31]. In brief, polyadenine was added to the 3′-end of RNAs using *E. coli* poly(A) polymerase (Enzynomics, Daejeon, Republic of Korea), and the polyadenylated RNAs were reverse-transcribed using M-MLV Reverse Transcriptase (Enzynomics) and an miR-RT-adapter-primer (Supplementary Table S1). To measure the mRNA transcript levels, RNA was first treated with DNase I (Enzynomics) to remove genomic DNA contaminants. RNA was then reverse-transcribed using M-MLV Reverse Transcriptase and random hexamers (Enzynomics). Quantitative PCR was performed using a BioFACT Real-Time PCR Master Mix (BIOFACT, Daejeon, Republic of Korea). To detect the isoforms of *fne* transcripts, RT-PCR was performed using a MegaFi Fidelity 2× PCR Master Mix (Applied Biological Materials, Richmond, BC, Canada) or BioFACT^TM^ 2× Taq PCR Master Mix (BIOFACT). PCR products were loaded into 2.5% agarose gel and then stained using SYBR^TM^ Safe (Thermo Fisher Scientific, Waltham, MA, USA). The band images were captured on a blue light transilluminator (miniPCR, Cambridge, MA, USA). The primer sequences used for semi- or quantitative-RT-PCR are listed in Supplementary Table S1.

### 2.5. Western Blotting

Western blot analysis was performed as previously described [32]. The following primer antibodies were used: anti-phospho-JNK (1:1000; Cell Signaling Technology, Danvers, MA, USA) and anti-β-Tubulin (1:5000; Developmental Studies Hybridoma Bank, University of Iowa, Iowa City, IA, USA). Chemiluminescence signals were detected using FluorChem HD2 (ProteinSimple, San Jose, CA, USA), and band intensities were quantified using ImageJ [33].

### 2.6. Body Weight and Wing Analysis

Groups of 8–10 adult flies of each genotype (3–5 days old) were transferred to new 1.5 mL tubes and weighed using an analytical balance (Mettler Toledo, Columbus, OH, USA). Four biological replicates per genotype and sex were analyzed.

The left wings of adult female (5 days old) were used for wing analysis. After capturing images using a stereomicroscope (Olympus), the relative wing size was measured using ImageJ software version 1.53 [33]. The wing cell size was analyzed as previously described [34]. The average wing cell size was calculated by dividing the specific wing area by the number of cells in that area. The total cell number of wings was estimated by calculating the wing size and total wing cell number.

### 2.7. Analysis of the Fat Body

To capture an image of the whole larval fat body, the fat body was dissected from five wandering third-instar larvae of each genotype and photographed using a stereomicroscope (Olympus).

Phalloidin staining was performed as previously described [35]. Briefly, fat bodies were dissected from wandering third-instar larvae and fixed with 4% paraformaldehyde (Electron Microscopy Sciences, Hatfield, PA, USA) in cold PBS for 20 min. After washing with cold PBS, fixed larval fat bodies were stained with Alexa Fluor 568-phalloidin (1:200; Molecular Probes, Eugene, OR, USA) or Phalloidin-iFluor 488 (1:500; Abcam, Cambridge, UK). The stained samples were placed in a mounting medium containing 4′,6-diamidino-2-phenylindole (DAPI; Abcam), and images were captured using a confocal laser microscope (Carl Zeiss, Oberkochen, Germany). The relative size of the phalloidin-stained cells was measured using ImageJ software version 1.53 [33].

### 2.8. Cell Culture

*Drosophila* S2 cells were maintained at 25 °C in Schneider’s insect medium (Thermo Fisher Scientific) supplemented with 10% fetal bovine serum (Welgene, Gyeongsan, Republic of Korea) and 100 U/mL penicillin–streptomycin (Welgene).

### 2.9. Luciferase Reporter Assay

To generate plasmid constructs for the reporter assay, the genomic region of the *fne* 3′-UTR was amplified by PCR. The amplified DNA fragment was then inserted downstream of the *Renilla* luciferase in the psiCHECK-2 vector (Promega, Madison, WI, USA). For the mutant form of the *fne* 3′-UTR, site-directed mutagenesis was performed using Phusion High-Fidelity DNA polymerase (Thermo Fisher Scientific), as previously described [36]. To generate the *miR-274*-overexpressing construct, a DNA fragment containing pre-miR-274 was amplified by PCR and cloned into the pMT/V5-His A vector (Invitrogen, Waltham, MA, USA). Both the miRNA-expressing and luciferase reporter constructs were co-transfected using *Trans*IT^®^-Insect Transfection Reagent (Mirus Bio, Madison, WI, USA). The activities of *Renilla* and firefly luciferase were determined using the Dual-Luciferase Reporter Assay System (Promega, Madison, WI, USA) 48 h after *miR-274* expression. The *Renilla* luciferase activity was normalized to the firefly luciferase activity. All primer sets for the reporter assay are listed in Supplementary Table S1.

## 3. Results

### 3.1. miR-274 Is Associated with Melanotic Mass Formation through the JNK—JAK/STAT Signaling Pathway Axis

**Figure 1:**
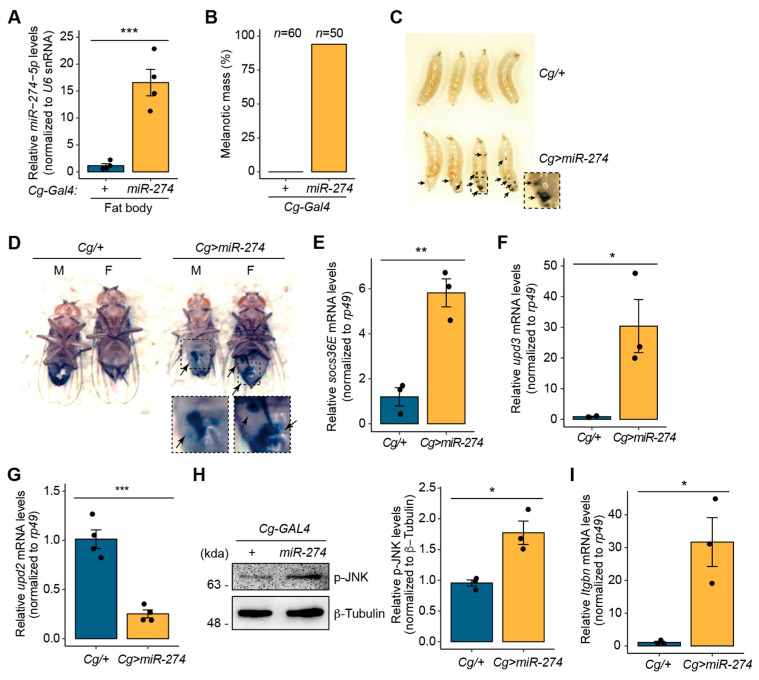
*miR-274* is involved in the formation of melanotic mass. (**A**) Overexpression of *miR-274-5p* in the fat body of *Cg>miR-274* larvae. *U6* snRNA was used as a control for normalization. (**B**) Quantitative data showing the percentage of wandering third-instar larvae with melanotic masses. *n* is the total number of analyzed larvae. (**C**) Wandering third-instar larvae of the indicated genotypes exhibiting melanotic masses. Melanotic masses are marked as arrows. The dashed box image is magnified. (**D**) Adult flies exhibiting melanotic masses in the indicated genotypes and sexes (M, male; F, female). Melanotic masses are marked as arrows. The dashed box images are magnified. (**E**) Expression level of *socs36E* mRNA in the fat body of *Cg>miR-274* larvae. *rp49* served as a control for normalization. (**F**) Expression level of *upd3* mRNA in the fat body of *Cg>miR-274* larvae. (**G**) Expression level of *upd2* mRNA in the fat body of *Cg>miR-274* larvae. (**H**) Protein level of p-JNK in the larval fat body of *Cg>miR-274*. Representative band image (left) and quantitative bar graph (right) are shown. β-Tubulin served as a loading control. (**I**) Expression level of *Itgbn* mRNA in the hemocytes of *Cg>miR-274* larvae. The error bars on the bar plots (**A**,**E**–**I**) indicate the standard error of the mean (SEM). The dots on the bar plot indicate individual data values. * *p* < 0.05, ** *p* < 0.01, and *** *p* < 0.001 compared with the control, as assessed by Student’s *t*-test.

The expression of *miR-274* exhibits dynamic changes during *Drosophila* development [32], and it is also upregulated under pathogen bacteria infection conditions [28]. Thus, we sought to investigate the biological roles of miR-274 under these upregulated conditions. To specially determine the biological roles of miR-274 in the fat bodies of *Drosophila,* we employed *Cg-GAL4* to overexpress miR-274 (hereafter, *Cg>miR-274*). *Cg-GAL4* is known to be active in fat bodies [37], where miR-274 is endogenously expressed (Supplementary Figure S1A). In the fat body of *Cg>miR-274* larvae, the expression of *miR-274-5p*, the major strand of miR-274, was significantly upregulated (Figure 1A). However, there was no significant increase in the expression level of *miR-274-3p* compared to the control (Supplementary Figure S1B). Interestingly, ninety-four percent of the *Cg>miR-274* larvae exhibited black masses throughout their bodies, whereas the *Cg/+* control larvae did not exhibit such melanotic masses (Figure 1B). The *Cg>miR-274* larvae had varying numbers and sizes of melanotic masses throughout their bodies (Figure 1C). These black masses persisted in the abdomens of both male and female flies (Figure 1D). In addition, we observed the formation of melanotic masses in the larvae when *miR-274* was overexpressed using another fat body-specific *GAL4* driver, *ppl-GAL4* (Supplementary Figure S1C). Taken together, these results suggest that miR-274 is associated with the formation of melanotic masses.

We proceeded to elucidate the molecular mechanisms underlying melanotic mass formation. According to previous reports, melanotic mass formation is strongly associated with the JAK/STAT signaling pathway [10]. Therefore, we sought to determine whether the overexpression of *miR-274* could alter the activity of the JAK/STAT signaling pathway. First, we measured the expression of *socs36E* mRNA, a target gene of the JAK/STAT signaling pathway [10,38], in the larval fat body of *Cg>miR-274*. Indeed, *socs36E* mRNA transcripts were significantly upregulated in the fat body of *Cg>miR-274* larvae compared to that in the control larval fat body (Figure 1E). These results suggest that the overexpression of *miR-274* leads to the activation of the JAK/STAT signaling pathway in the fat body.

Next, we wondered how miR-274 could affect the JAK/STAT signal pathway. Thus, we determined the mRNA transcript levels of *upds*, a ligand of the JAK/STAT signaling pathway [39,40], in the larval fat body of *Cg>miR-274*. Interestingly, we found a significant upregulation of *upd3* mRNA transcripts in the fat body of *Cg>miR-274* larvae, with an approximately 30.4-fold increase compared to that in the controls (Figure 1F). In contrast, the expression of *upd2* was downregulated with a relatively smaller change of approximately 4.0-fold in the fat body of *Cg>miR-274* larvae compared to that in the controls (Figure 1G). Furthermore, no expression of *upd1* was detected in the fat body. These data indicate that the overexpression of miR-274 leads to a significant upregulation of *upd3,* which activates the JAK/STAT signaling pathway.

The expression of the *upd3* cytokine is induced by the JNK signaling pathway [11]. Thus, to examine whether miR-274 is linked to the activation of the JNK signaling pathway, we determined the level of active phospho-JNK (p-JNK) in the fat body of *Cg>miR-274* larvae. The p-JNK level was found to increase in the larval fat body of *Cg>miR-274* relative to that in the control fat body (Figure 1H, Supplementary Figure S2), suggesting that *miR-274* overexpression also activates the JNK signaling pathway.

Activation of the JNK signaling pathway in other tissues can upregulate the expression of *upd3*, which triggers a systemic response in hemocytes. This response can increase hemocyte proliferation and lamellocyte differentiation, ultimately resulting in melanotic mass formation [10,11]. Therefore, to investigate whether the population of lamellocytes, which are closely associated with melanotic mass, is increased in the hemolymph of *Cg>miR-274* larvae, we determined the level of *Integrin betanu subunit* (*Itgbn*), which is a marker gene of lamellocytes [41] using RT-qPCR. Remarkably, the expression level of *Itgbn* was significantly higher in the hemocytes of *Cg>miR-274* larvae than in the control (Figure 1I), suggesting that lamellocytes were more differentiated in the hemolymph of *Cg>miR-274* larvae. Collectively, our data suggest that miR-274 is associated with melanotic mass formation by regulating the JNK-JAK/STAT pathway axis under its upregulated conditions.

### 3.2. Fat Body Overexpression of miR-274 Leads to Growth Reduction through Defects in the Fat Body

**Figure 2:**
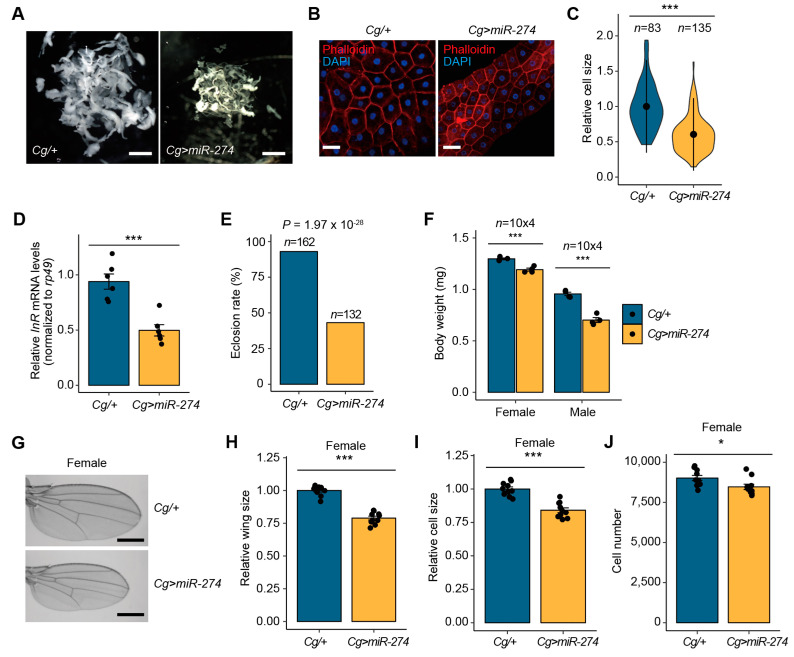
Overexpression of *miR-274* in the fat body reduces developmental growth. (**A**) Whole fat bodies of wandering third-instar larvae of the indicated genotypes (*n* = 5). Scale bar, 1 mm. (**B**) Representative phalloidin staining images of the larval fat body (phalloidin, red; DAPI, blue). Scale bar, 50 µm. (**C**) Relative size of the fat body cells in *Cg>miR-274* larvae. The quantitative violin plot is shown as the mean ± standard deviation (SD). *n* is the total number of analyzed fat body cells (from five larvae). (**D**) Expression level of *InR* mRNA in the fat body of *Cg>miR-274* larvae. (**E**) Eclosion rate from pupae to adult flies in each genotype. *n* is the total number of analyzed pupae. *P*-value, as assessed by Chi-square test. (**F**) Body weight of *Cg>miR-274* flies. *n* is the total number of analyzed flies. (**G**) Representative wing images of female flies of the indicated genotypes. Scale bar, 0.5 mm. (**H**) Relative comparison of wing size between *Cg/+* and *Cg>miR-274* female flies (*n* = 10 per each genotype). (**I**,**J**) Relative cell size (**I**) and total cell number (**J**) of adult wings analyzed in the H panel. All bar plots (**D**,**F**,**H**–**J**) are shown as the mean ± SEM. The dots on the bar plot indicate individual data values. * *p* < 0.05 and *** *p* < 0.001 compared with the control, as assessed by Student’s *t*-test.

We additionally investigated the phenotypic consequences observed in the fat bodies of *Cg>miR-274* larvae. Interestingly, the overall size of the fat bodies expressing *miR-274* was markedly reduced compared to that of the control fat bodies (Figure 2A). Moreover, the size of cells in the fat body was notably reduced in *Cg>miR-274* larvae compared to that in the control larvae (Figure 2B,C). These data indicate that the overexpression of *miR-274* causes a reduction in the tissue growth of the larval fat body in addition to melanotic mass formation. Activation of the JAK/STAT signaling pathway is linked to the downregulation of *Insulin-like receptor* (*InR*) mRNA expression [42]. Because the JAK/STAT signaling activation was observed in the fat body of *Cg>miR-274* larvae, we sought to determine whether the expression of *InR* mRNA is changed in the fat body by *miR-274* overexpression. As expected, the expression of *InR* mRNA was reduced in the fat body of *Cg>miR-274* larvae compared with the controls (Figure 2D), indicating that a reduced size of the fat body by *miR-274* overexpression may be likely associated with a decrease in *InR* mRNA expression.

As the fat body is a crucial tissue associated with energy metabolism and growth [43], we continuously monitored the effects of *miR-274* overexpression on developmental growth when *miR-274* was overexpressed in the fat body. Most *Cg>miR-274* larvae developed into the pupal stage; however, more than 50% of these pupae could not eclose into adult flies (Figure 2E), indicating that miR-274-induced defects in tissue growth of the larval fat body affect normal development.

Interestingly, the growth of *Cg>miR-274* adult flies was reduced relative to that of control flies. To further explore this reduction in growth, we compared the body weights of 3–5-day-old flies between *Cg/+* and *Cg>miR-274*. In both males and females, we observed a significant reduction in the body weight of *Cg>miR-274* flies (8.1%–26.5%) compared to *Cg/+* control flies (Figure 2F). Furthermore, in proportion to body weight, the size of *Cg>miR-274* adult wings remarkably decreased relative to the size of control wings (Figure 2G,H). To determine whether the reduction in whole wing size was due to the size and/or number of wing cells, we analyzed the cell size and number of wings in the indicated genotypes. Both the cell size and number of wings of *Cg>miR-274* flies were lower than those in the control flies (Figure 2I,J). Taken together, considering previous results of the upregulation of *miR-274* at the larval-to-pupal transitions [32], these results indicate that the untimely upregulation of *miR-274* results in the inhibition of growth through the reduction in total tissue mass in the fat body.

### 3.3. miR-274 Negatively Regulates the Expression of Fne

**Figure 3:**
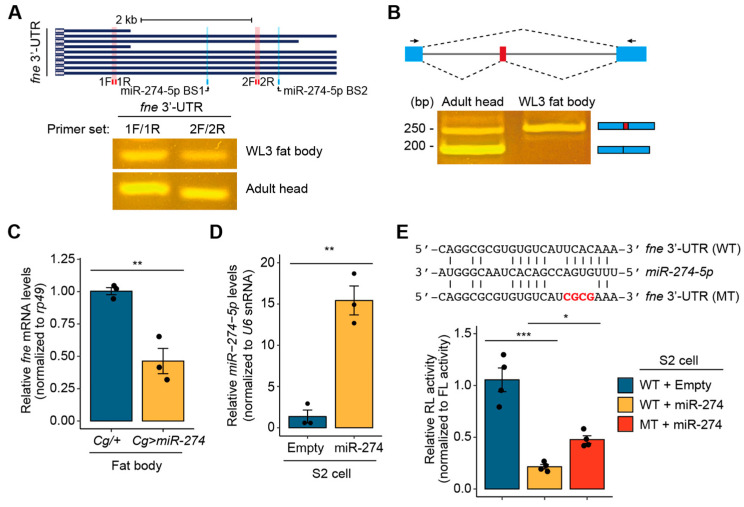
*miR-274* suppresses *fne* expression. (**A**) Expression of the *fne* mRNA transcripts with the short or extended 3′-UTR. Isoforms of *fne* mRNA transcripts with different lengths of 3′-UTR, each primer binding site (1F/1R and 2F/2R, red box), and two miR-274-5p binding sites (BS1 and BS2, blue box) are shown (top). Semi-RT-qPCR at two different sites in the *fne* 3′-UTR in the larval fat bodies (bottom). Adult heads were used as a positive control. (**B**) Expression of nuclear *fne* (*nfne*) transcript containing the microexon (red box) in the larval fat bodies. Schematic of the *fne* microexon (top) and the expression of two *fne* isoforms in the larval fat bodies and adult heads (bottom). Blue and red boxes indicate exons, and gray lines indicate introns. The primer binding sites for RT-qPCR are marked as arrows. (**C**) Downregulation of the *fne* mRNA level in the fat body of *Cg>miR-274* larvae. *rp49* served as a control for normalization. (**D**) Overexpression of *miR-274-5p* in S2 cells. *U6* snRNA was used as a control. (**E**) Relative activity of the *Renilla*-luciferase (RL) fused with either the wild-type (WT) or mutated (MT) *fne* 3′-UTR. The sequences of WT and MT *fne* 3′-UTR and miR-274-5p are shown (top). The mutated sequences are highlighted in bold red. The RL activity was normalized to the firefly luciferase (FL) activity (bottom). All bar plots are shown as the mean ± SEM. The dots on the bar plot indicate individual data values. * *p <* 0.05, ** *p* < 0.01, and *** *p <* 0.001 compared with the control, as assessed by Student’s *t*-test (**C**,**D**) or ANOVA with a supplementary Dunnett’s test (**E**).

We wondered how miR-274 controls melanotic mass formation and growth and thus investigated the regulatory mechanism of miR-274. Using the miRNA target prediction tool, TargetScanFly [9], we first searched for potential target genes that could be regulated by miR-274-5p, which is the main strand of miR-274. We found 173 transcripts with conserved miR-274-5p binding sites (Supplementary Table S2). Among these, based on a screening study of the gene network regulating blood cell homeostasis in *Drosophila* [2], we selected *found in neurons* (*fne*) that encodes an RNA-binding protein associated with the regulation of mRNA processing, such as splicing and alternative polyadenylation (APA) of mRNA [26,44]. According to the prediction using TargetScanFly, miR-274-5p could be bound to two potential sites in the *fne* 3′-UTR: one conserved site (miR-274-5p BS1) and one poorly conserved site (miR-274-5p BS2) (Figure 3A, top).

In *Drosophila*, *fne* mRNAs are mainly expressed in the nervous system and exist as several isoforms with different lengths of the 3′-UTR [26]. Thus, we determined whether *fne* mRNA is also expressed in the larval fat body in addition to the nervous system. By semi-RT-qPCR using two different primer sets, we detected *fne* mRNA with a short and extended 3′-UTR in the fat body of wandering third-instar larvae and adult heads. Consistent with previous results [24,26], the *fne* mRNA transcript with an extended 3′-UTR was detected in adult heads and was also expressed in the fat body (Figure 3A, bottom; Supplementary Figure S2). Under Elav-depleted conditions, the nucleus-localized Fne (nFne) bearing the microexon can be induced, which regulates the APA process [26,44]. Therefore, we investigated whether *nfne* is expressed in the fat body, which is a tissue that does not express Elav. Interestingly, we found that only the *nfne* transcript containing the microexon was expressed in the larval fat bodies, whereas both general *fne* and *nfne* transcripts were expressed at high and low levels, respectively, in adult heads (Figure 3B, Supplementary Figure S2). These findings suggest that the APA-related nFne likely functions in the larval fat body.

We next examined whether miR-274 negatively regulates the expression of *fne* in larval fat bodies. Accordingly, we determined the levels of *fne* mRNA transcripts in the fat bodies of *Cg/+* and *Cg>miR-274* larvae. As expected, the expression of *fne* mRNA was significantly reduced in the larval fat bodies overexpressing *miR-274* compared to that in the control fat bodies (Figure 3C). These data support the hypothesis that miR-274 suppresses *fne* mRNA expression in the larval fat bodies.

To clarify the regulatory interaction between miR-274 and *fne* mRNA, we performed a luciferase reporter assay. When *miR-274* was overexpressed in S2 cells, the activity of *Renilla* luciferase (RL) fused with wild-type *fne* 3′-UTR significantly decreased (Figure 3D,E). In contrast, the inhibitory activity of *miR-274* was partially reduced upon co-expression of RL fused with the *fne* 3′-UTR containing a mutation in the conserved miR-274-5p binding site (miR-274-5p BS1) (Figure 3E). Taken together, our results suggest that miR-274-5p directly suppresses *fne* expression in *Drosophila*.

### 3.4. Fne Is Involved in Melanotic Mass Formation as the Biological Target of miR-274

**Figure 4:**
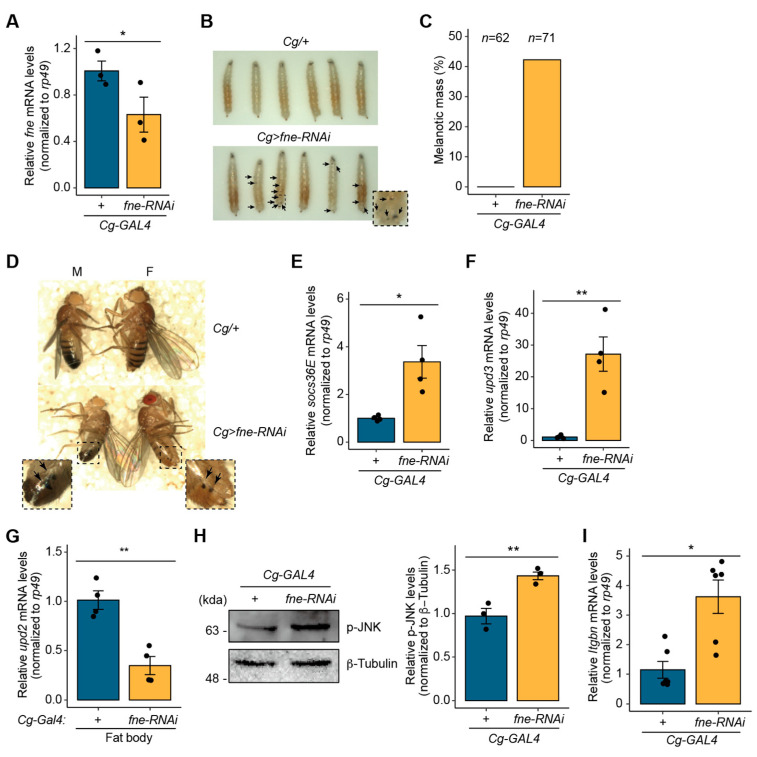
Depletion of *fne* results in melanotic mass formation. (**A**) Knockdown of *fne* in the fat body using RNAi^TRiP^ line driven by *Cg-GAL4*. *rp49* served as a control for normalization. (**B**) Melanotic mass formation in *Cg>fne-RNAi* larvae. Melanotic masses are marked as arrows. The dashed box image is magnified. (**C**) Percentage of *Cg>fne-RNAi* larvae exhibiting melanotic masses. *n* is the total number of analyzed larvae. (**D**) Maintenance of melanotic mass in *Cg>fne-RNAi* adults. Melanotic masses are marked as arrows. The dashed box images are magnified. (**E**) Expression level of *socs36E* mRNA in the fat body of *Cg>fne-RNAi* larvae. (**F**) Expression level of *upd3* mRNA in the fat body of *Cg>fne-RNAi* larvae. (**G**) Expression level of *upd2* mRNA in the fat body of *Cg>fne-RNAi* larvae. (**H**) Protein level of p-JNK in the larval fat body of *Cg>fne-RNAi*. Representative band image (left) and quantitative bar graph (right) are shown. β-Tubulin served as a loading control. (**I**) Expression level of *Itgbn* mRNA in the hemocytes of *Cg>fne-RNAi*. The error bars on the bar plots (**A**,**E**–**I**) indicate SEM. The dots on the bar plot indicate individual data values. * *p* < 0.05 and ** *p* < 0.01 compared with the control, as assessed by Student’s *t*-test.

As *fne* is suppressed by miR-274 as a direct target in *Drosophila*, we sought to determine whether the loss of *fne* leads to phenotypic consequences similar to the defects caused by miR-274 driven by *Cg-GAL4*. The previous RNAi screening study reported that RNA splicing-associated protein *fne* may be associated with melanotic mass formation [2]. To test this, we first knocked down *fne* expression in the fat body using *Cg-GAL4* (*Cg>fne-RNAi*) (Figure 4A). Consistent with the results for *Cg>miR-274* larvae, we observed that melanotic masses remarkably appeared throughout the body of *Cg>fne-RNAi* larvae (Figure 4B), and 42.3% of the *Cg>fne-RNAi* larvae exhibited melanotic mass formation (Figure 4C). Melanotic masses also persisted in both sexes into adulthood (Figure 4D). These findings suggest that the loss of *fne* is associated with melanotic mass formation.

We next examined whether the depletion of *fne* causes an increase in the activity of the JAK/STAT signaling pathway in the larval fat body, similar to *Cg>miR-274* larvae. The expression level of *socs36E* was significantly higher in the fat bodies of *Cg>fne-RNAi* larvae than in the control fat bodies (Figure 4E), indicating that the depletion of *fne* activates the JAK/STAT signaling pathway. Furthermore, the expression of *upd3* was markedly upregulated in the fat bodies of *Cg>fne-RNAi* larvae with an approximately 27.2-fold increase compared to that in the controls (Figure 4F). In contrast, the expression of *upd2* was downregulated, with an approximately 2.9-fold decrease compared to that in the controls (Figure 4G). These results suggest that *fne* is involved in the activation of the JAK/STAT signaling pathway through a significant upregulation of *upd3* expression rather than *upd2* expression.

Furthermore, we determined whether *fne* alters the level of active p-JNK, as observed with *miR-274* overexpression. The level of active p-JNK increased in the fat body of *Cg> fne-RNAi* larvae compared to that in the control (Figure 4H, Supplementary Figure S2), indicating that *fne* depletion activates the JNK signaling pathway.

We investigated whether the reduction in *fne* expression leads to increased lamellocyte differentiation. We determined the levels of *Itgbn* mRNA in the hemolymph of *Cg>fne-RNAi* larvae. Consistent with *Cg>miR-274* larvae, *Itgbn* mRNA levels were higher in the hemocytes of *Cg>fne-RNAi* larvae than in that of the controls (Figure 4I). Thus, the results imply that the depletion of *fne* causes an increase in the number of lamellocytes. Collectively, our data suggest that *fne*, as a biological target of miR-274, is associated with melanotic mass formation through the JNK–JAK/STAT signaling pathway.

### 3.5. Fne Depletion Leads to Growth Reduction

**Figure 5:**
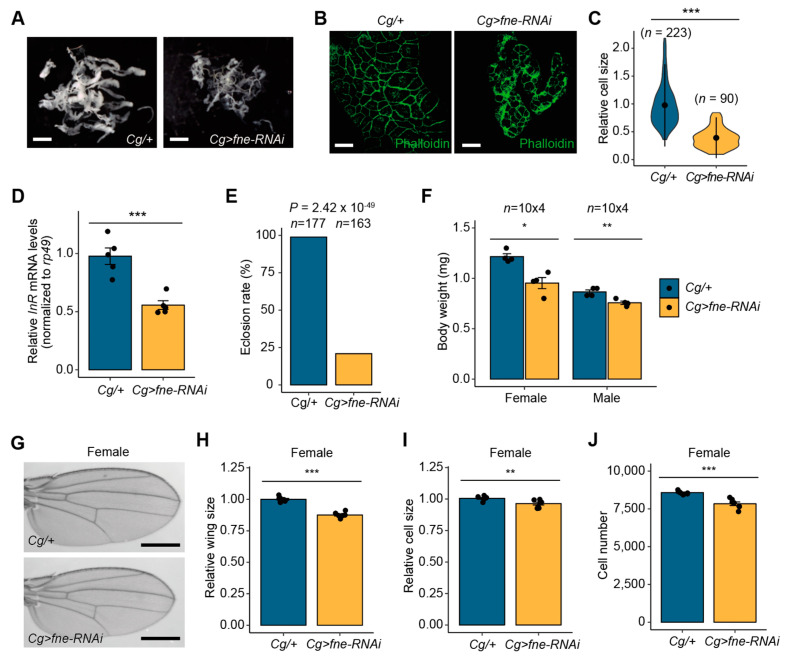
Knockdown of *fne* causes growth reduction. (**A**) Whole fat bodies of wandering third-instar larvae of the indicated genotypes (*n* = 5). Scale bar, 1 mm. (**B**) Representative phalloidin staining images of the larval fat body (phalloidin, green). Scale bar, 50 µm. (**C**) Relative size of the fat body cells in *Cg>fne-RNAi* larvae. The quantitative violin plot is shown as the mean ± standard deviation (SD). *n* is the total number of analyzed fat body cells (from five larvae). (**D**) Expression level of *InR* mRNA in the fat body of *Cg>fne-RNAi* larvae. (**E**) Reduced eclosion rate of *Cg>fne-RNAi* pupae. *n* is the total number of analyzed pupae. *p*-value, as assessed by Chi-square test. (**F**) Body weight of *Cg>fne-RNAi* flies. *n* is the total number of analyzed flies. (**G**) Representative wing images of female flies of the indicated genotypes. Scale bar, 0.5 mm. (**H**) Relative comparison of wing size between *Cg/+* and *Cg>fne-RNAi* female flies (*n* = 7 per each genotype). (**I**,**J**) Relative cell size (**I**) and total cell number (**J**) of adult wings analyzed in the H panel. All bar plots (**D**,**F**,**H**–**J**) are shown as the mean ± SEM. The dots on the bar plot indicate individual data values. * *p* < 0.05, ** *p* < 0.01, and *** *p* < 0.001 compared with the control, as assessed by Student’s *t*-test.

We investigated whether *fne* is also implicated in developmental growth, similar to miR-274. First, we analyzed the total fat body mass in *Cg>fne-RNAi* larvae. Similar to *Cg>miR-274* larvae, the overall size of the fat body was remarkably reduced in *Cg>fne-RNAi* larvae compared to that in control larvae (Figure 5A). To further examine this phenotype, we compared the sizes of fat body cells between *Cg/+* and *Cg>fne-RNAi* larvae after F-actin staining with phalloidin. The cell size of the fat body in *Cg>fne-RNAi* larvae was significantly smaller than that in the controls (Figure 5B,C). In addition, consistent with the observations from the fat body of *Cg>miR-274* larvae, the expression of *InR* mRNA was significantly reduced in the fat body of *Cg>fne-RNAi* larvae compared with the controls (Figure 5D). These results suggest that the reduced fat body resulting from *fne* depletion may be likely associated with a decrease in *InR* mRNA expression.

The eclosion rate of *Cg>fne-RNAi* pupae was remarkably reduced compared to that of control pupae (Figure 5E). To investigate whether defects in the larval fat body of *Cg>fne-RNAi* affected developmental growth, we measured the body weights of *Cg>fne-RNAi* flies. Consistent with the reduction in body weight in *Cg>miR-274* flies, *Cg>fne-RNAi* flies of both sexes displayed significantly lower body weight than control flies (Figure 5F). Moreover, we observed a decrease in the whole size of *Cg>fne-RNAi* wings relative to control wings in proportion to body weight (Figure 5G,H). The size and the total number of wing cells in *Cg>fne-RNAi* flies were reduced compared to those in control flies (Figure 5I,J). Together, these observations indicate that *fne*, a target of miR-274, also plays a role in developmental growth.

## 4. Discussion

In this study, we demonstrated that miR-274 activates the JNK–JAK/STAT signaling axis by targeting *fne*, which in turn controls the formation of melanotic mass. However, further studies are needed to address the detailed mechanism by which *fne* controls the JNK–JAK/STAT signaling pathways. Based on previous reports [26], Fne may regulate gene expression through its involvement as an RNA-binding protein in the APA process. In particular, the nucleus-localized nFne is involved in this process under Elav-depleted conditions [26,44]. Based on our results, the nFne isoform is likely functional in the APA process in the larval fat body, where Elav is not expressed. Thus, the depletion of *fne* may induce a shortened 3′-UTR of *fne*-target mRNAs, thereby reducing the chance of negative regulation by miRNAs; this is because miRNAs mainly suppress the expression of target genes by binding to the 3′-UTR of mRNAs [14], which may result in an increase in the expression of genes targeted by Fne. By the targets of RNA-binding proteins identified by editing (TRIBE) method [45], target mRNAs directly bound by Fne have been identified in *Drosophila* S2 cells [46]. The identified genes include *Stat92E*, a key factor in the JAK/STAT signaling pathway, and positive regulators of JNK signaling, such as *Cell division cycle 42* (*Cdc42*), *Rac1*, and *cryptocephal* (*crc*). Thus, changes in the length of the 3′-UTR of these genes involved in the JNK or the JAK/STAT signaling pathway, which are regulated by Fne, may alter their expression level.

Our findings indicate that miR-274 activates JNK signaling by suppressing *fne*, which in turn induces the expression of *upd3* in the fat body. Subsequently, Upd3 derived from the fat body may stimulate lamellocyte differentiation, triggering melanotic mass formation. Although we provided evidence of the association between miR-274 overexpression in the fat body and melanotic mass formation based on observations using *Cg-* and *ppl-GAL4* drivers, we cannot completely exclude the possibility that miR-274 may regulate the JNK–JAK/STAT signaling pathway in blood cells in addition to the fat body. The *Cg-GAL4* driver primarily activates the fat body but also drives gene expression in blood cells. [37]. Furthermore, the effects of miR-274 on melanotic mass formation were relatively weaker in *ppl>miR-274* larvae compared to *Cg>miR-274* larvae. Thus, interactions between the regulatory networks of the fat body and hemocytes may synergistically influence melanotic mass formation.

We noted a significant reduction in tissue size and activation of the JNK–JAK/STAT signaling pathway in the fat body when *miR-274* and *fne* were overexpressed and depleted, respectively. In addition to JAK/STAT signaling, JNK signaling is well known to play a pro-apoptotic role in cell death and negatively regulates insulin/IGF-like signaling in both mammals and *Drosophila* [47,48]. The activation of JNK signaling has been demonstrated to cause defects in the normal growth and development of the wing and eye in *Drosophila* [49,50]. These findings provide support for the association between miR-274, *fne*, and developmental growth. In addition, a previous report described that knockdown of *upd2* in the fat body, but not *upd1* and *upd3*, leads to growth reduction by regulating the secretion of *Drosophila* insulin-like peptides in the insulin-producing cells [51]. Therefore, as observed in this study, the downregulation of *upd2* in the fat body may affect growth reduction.

According to previous reports, the expression of *pre-miR-274* is higher in the glia than in other tissues during the larval stage of *Drosophila* [27]. However, mature-miR-274 is released as an exosome and broadly distributed to other cells, including synaptic boutons, muscle cells, and tracheal cells [27]. This finding implies that glia-derived exosomal mature-miR-274 could circulate in the larval hemolymph and affect the expression of its target genes in several target tissues, such as hemocytes and fat bodies, along with miR-274 transcribed in the tissue itself. The biological target gene of miR-274, *fne,* is primarily expressed in neuronal tissue in *Drosophila* [24]. The expression of both *miR-274* and *fne* in the same tissues indicates that they are likely to maintain a regulatory network, which is similar to our findings in fat bodies. Based on our previous results, the expression of *miR-274* is upregulated during the larval-to-pupal transition [32], which suggests that changes in *miR-274* expression may alter the composition of the extended 3′-UTR of neuronal-specific genes by regulating the expression of *fne* during this developmental stage. Thus, future studies should investigate the perturbation of APA in neuron-specific genes during metamorphosis.

We observed a reduction in both *fne* mRNA and reporter activity with the *fne* 3′-UTR when miR-274 was overexpressed. miR-274-5p was found to negatively regulate the expression of *fne* at the transcript level by binding to at least one conserved site (miR-274-5p BS1) in the 3′-UTR of *fne* mRNA. However, the luciferase reporter activity suppressed by miR-274 was found to be partially restored when the region corresponding to the miR-274-5p seed was mutated in the miR-274-5p BS1. This result implies that other binding sites may exist for miR-274-5p in the *fne* 3′-UTR. The poorly conserved binding site (miR-274-5p BS2) in the *fne* 3′-UTR could be a potential regulatory binding site for miR-274-5p. Future studies could examine whether miR-274-5p targets miR-274-5p BS2 in the *fne* 3′-UTR.

A relatively stronger formation of melanotic masses was observed in larvae overexpressing miR-274 than in *fne*-depleted larvae. Most *Cg>miR-274* larvae exhibited a melanotic mass, whereas less than 50% *of Cg>fne-RNAi* larvae had black dots. The melanotic masses in the individual *Cg>miR-274* larvae were numerous and larger than those in the *Cg>fne-RNAi* larvae. Such small differences between the lines linked in the regulatory network may arise from other genes affected by alterations in *miR-274* or *fne* expression. TargetScanFly identified 173 potential miR-274-5p targets. Some of these targets, such as *S-adenosylmethionine Synthetase* (*Sam-S*) and *slimfast* (*slif*), might directly or indirectly affect the JNK and/or JAK/STAT signaling pathways. In addition, although miR-274-3p is not the predominant strand of miR-274, miR-274-3p might contribute to melanotic mass formation and/or growth. TargetScanFly identified numerous potential target genes of miR-274-3p. For example, GO term analysis using DAVID [52,53] revealed 11 genes (*Connector of kinase to AP-1*, *Menin 1*, *Protein phosphatase V*, *Ras-like protein A*, *Striatin interacting protein*, *alphabet*, *capping protein alpha*, *fiery mountain*, *flapwing*, *icarus*, and *pebbled*) that can play negative regulators of the JNK signaling pathway.

Collectively, our findings provide insights into the potential molecular mechanisms underlying these phenomena. Further studies should explore these mechanisms in detail to provide a more comprehensive understanding of the physiological processes.

## 5. Conclusions

In this study, under its overexpression conditions, *Drosophila* miR-274 was found to play a regulatory role in the JNK and JAK/STAT signaling pathways by suppressing the expression of *fne* encoding an RNA-binding protein that functions in RNA processing, such as APA and alternative splicing. This regulatory network of miR-274–*fne*–JNK signaling in the larval fat body is associated with the formation of melanotic mass and developmental growth. Thus, our findings provide valuable insights into the molecular mechanisms underlying melanotic mass formation and growth. Furthermore, a better understanding of this regulatory mechanism will contribute to the development of novel ideas for regulating immune defense responses against invading pathogens and growth regulation.

## Data Availability

Datasets are available upon request. The raw data supporting the conclusions of this article will be made available by the authors without any reservations.

## Supplementary Materials

The following supporting information can be downloaded at: https://www.mdpi.com/article/10.3390/insects14080709/s1. Supplementary Table S1: Sequences of oligonucleotides used in this study. Supplementary Table S2: Potential target genes predicted using TargetScanFly. Supplementary Figure S1: Expression of *miR-274*, and the formation of melanotic masses in *ppl>miR-274* larvae. Supplementary Figure S2: Images of Western blotting and DNA gel presented in the main figures.

## References

[1] Hartenstein V. (2006). Blood cells and blood cell development in the animal kingdom. Annu. Rev. Cell Dev. Biol..

[2] Avet-Rochex A., Boyer K., Polesello C., Gobert V., Osman D., Roch F., Auge B., Zanet J., Haenlin M., Waltzer L. (2010). An in vivo RNA interference screen identifies gene networks controlling *Drosophila melanogaster* blood cell homeostasis. BMC Dev. Biol..

[3] Lanot R., Zachary D., Holder F., Meister M. (2001). Postembryonic hematopoiesis in *Drosophila*. Dev. Biol..

[4] Tepass U., Fessler L.I., Aziz A., Hartenstein V. (1994). Embryonic origin of hemocytes and their relationship to cell death in *Drosophila*. Development.

[5] Crozatier M., Meister M. (2007). *Drosophila* haematopoiesis. Cell Microbiol..

[6] Kocks C., Cho J.H., Nehme N., Ulvila J., Pearson A.M., Meister M., Strom C., Conto S.L., Hetru C., Stuart L.M. (2005). Eater, a transmembrane protein mediating phagocytosis of bacterial pathogens in *Drosophila*. Cell.

[7] Ramet M., Pearson A., Manfruelli P., Li X., Koziel H., Gobel V., Chung E., Krieger M., Ezekowitz R.A. (2001). *Drosophila* scavenger receptor CI is a pattern recognition receptor for bacteria. Immunity.

[8] Minakhina S., Steward R. (2006). Melanotic mutants in *Drosophila*: Pathways and phenotypes. Genetics.

[9] Agarwal V., Subtelny A.O., Thiru P., Ulitsky I., Bartel D.P. (2018). Predicting microRNA targeting efficacy in *Drosophila*. Genome Biol..

[10] Myllymaki H., Ramet M. (2014). JAK/STAT pathway in *Drosophila* immunity. Scand. J. Immunol..

[11] Pastor-Pareja J.C., Wu M., Xu T. (2008). An innate immune response of blood cells to tumors and tissue damage in *Drosophila*. Dis. Model. Mech..

[12] Boulan L., Milan M., Leopold P. (2015). The Systemic Control of Growth. Cold Spring Harb. Perspect. Biol..

[13] Colombani J., Raisin S., Pantalacci S., Radimerski T., Montagne J., Leopold P. (2003). A nutrient sensor mechanism controls *Drosophila* growth. Cell.

[14] He L., Hannon G.J. (2004). MicroRNAs: Small RNAs with a big role in gene regulation. Nat. Rev. Genet..

[15] Kadener S., Rodriguez J., Abruzzi K.C., Khodor Y.L., Sugino K., Marr M.T., Nelson S., Rosbash M. (2009). Genome-wide identification of targets of the drosha-pasha/DGCR8 complex. RNA.

[16] Lee Y.S., Nakahara K., Pham J.W., Kim K., He Z., Sontheimer E.J., Carthew R.W. (2004). Distinct roles for *Drosophila* Dicer-1 and Dicer-2 in the siRNA/miRNA silencing pathways. Cell.

[17] Hammond S.M. (2005). Dicing and slicing: The core machinery of the RNA interference pathway. FEBS Lett..

[18] Filipowicz W., Bhattacharyya S.N., Sonenberg N. (2008). Mechanisms of post-transcriptional regulation by microRNAs: Are the answers in sight?. Nat. Rev. Genet..

[19] Kozomara A., Birgaoanu M., Griffiths-Jones S. (2019). miRBase: From microRNA sequences to function. Nucleic Acids Res..

[20] Colaianni D., De Pitta C. (2022). The Role of microRNAs in the *Drosophila melanogaster* Visual System. Front. Cell Dev. Biol..

[21] Luo W., Sehgal A. (2012). Regulation of circadian behavioral output via a MicroRNA-JAK/STAT circuit. Cell.

[22] Yoon W.H., Meinhardt H., Montell D.J. (2011). miRNA-mediated feedback inhibition of JAK/STAT morphogen signalling establishes a cell fate threshold. Nat. Cell Biol..

[23] Wang Z., Xia X., Li J., Igaki T. (2022). Tumor elimination by clustered microRNAs miR-306 and miR-79 via noncanonical activation of JNK signaling. eLife.

[24] Zanini D., Jallon J.M., Rabinow L., Samson M.L. (2012). Deletion of the *Drosophila* neuronal gene found in neurons disrupts brain anatomy and male courtship. Genes Brain Behav..

[25] Zaharieva E., Haussmann I.U., Brauer U., Soller M. (2015). Concentration and Localization of Coexpressed ELAV/Hu Proteins Control Specificity of mRNA Processing. Mol. Cell Biol..

[26] Wei L., Lee S., Majumdar S., Zhang B., Sanfilippo P., Joseph B., Miura P., Soller M., Lai E.C. (2020). Overlapping Activities of ELAV/Hu Family RNA Binding Proteins Specify the Extended Neuronal 3′ UTR Landscape in *Drosophila*. Mol. Cell.

[27] Tsai Y.W., Sung H.H., Li J.C., Yeh C.Y., Chen P.Y., Cheng Y.J., Chen C.H., Tsai Y.C., Chien C.T. (2019). Glia-derived exosomal miR-274 targets Sprouty in trachea and synaptic boutons to modulate growth and responses to hypoxia. Proc. Natl. Acad. Sci. USA.

[28] Wei G., Sun L., Li R., Li L., Xu J., Ma F. (2018). Dynamic miRNA-mRNA regulations are essential for maintaining *Drosophila* immune homeostasis during Micrococcus luteus infection. Dev. Comp. Immunol..

[29] Kim M.J., Choe K.M. (2014). Basement membrane and cell integrity of self-tissues in maintaining *Drosophila* immunological tolerance. PLoS Genet..

[30] Lim D.H., Oh C.T., Han S.J., Lee Y.S. (2014). Methods for studying the biological consequences of endo-siRNA deficiency in *Drosophila melanogaster*. Methods Mol. Biol..

[31] Lim J.H., Kim D.J., Lee D.E., Han J.Y., Chung J.H., Ahn H.K., Lee S.W., Lim D.H., Lee Y.S., Park S.Y. (2015). Genome-wide microRNA expression profiling in placentas of fetuses with Down syndrome. Placenta.

[32] Lim D.H., Lee S., Choi M.S., Han J.Y., Seong Y., Na D., Kwon Y.S., Lee Y.S. (2020). The conserved microRNA miR-8-3p coordinates the expression of V-ATPase subunits to regulate ecdysone biosynthesis for *Drosophila* metamorphosis. FASEB J..

[33] Schneider C.A., Rasband W.S., Eliceiri K.W. (2012). NIH Image to ImageJ: 25 years of image analysis. Nat. Methods.

[34] McCabe J., French V., Partridge L. (1997). Joint regulation of cell size and cell number in the wing blade of *Drosophila melanogaster*. Genet. Res..

[35] Lim D.H., Lee S., Han J.Y., Choi M.S., Hong J.S., Lee Y.S. (2019). MicroRNA miR-252 targets mbt to control the developmental growth of *Drosophila*. Insect Mol. Biol..

[36] Edelheit O., Hanukoglu A., Hanukoglu I. (2009). Simple and efficient site-directed mutagenesis using two single-primer reactions in parallel to generate mutants for protein structure-function studies. BMC Biotechnol..

[37] Asha H., Nagy I., Kovacs G., Stetson D., Ando I., Dearolf C.R. (2003). Analysis of Ras-induced overproliferation in *Drosophila* hemocytes. Genetics.

[38] Callus B.A., Mathey-Prevot B. (2002). SOCS36E, a novel *Drosophila* SOCS protein, suppresses JAK/STAT and EGF-R signalling in the imaginal wing disc. Oncogene.

[39] Wright V.M., Vogt K.L., Smythe E., Zeidler M.P. (2011). Differential activities of the *Drosophila* JAK/STAT pathway ligands Upd, Upd2 and Upd3. Cell Signal.

[40] Agaisse H., Petersen U.M., Boutros M., Mathey-Prevot B., Perrimon N. (2003). Signaling role of hemocytes in *Drosophila* JAK/STAT-dependent response to septic injury. Dev. Cell.

[41] Bazzi W., Cattenoz P.B., Delaporte C., Dasari V., Sakr R., Yuasa Y., Giangrande A. (2018). Embryonic hematopoiesis modulates the inflammatory response and larval hematopoiesis in *Drosophila*. eLife.

[42] Shin M., Cha N., Koranteng F., Cho B., Shim J. (2020). Subpopulation of Macrophage-Like Plasmatocytes Attenuates Systemic Growth via JAK/STAT in the *Drosophila* Fat Body. Front. Immunol..

[43] Edgar B.A. (2006). How flies get their size: Genetics meets physiology. Nat. Rev. Genet..

[44] Carrasco J., Mateos F., Hilgers V. (2022). A critical developmental window for ELAV/Hu-dependent mRNA signatures at the onset of neuronal differentiation. Cell Rep..

[45] McMahon A.C., Rahman R., Jin H., Shen J.L., Fieldsend A., Luo W., Rosbash M. (2016). TRIBE: Hijacking an RNA-Editing Enzyme to Identify Cell-Specific Targets of RNA-Binding Proteins. Cell.

[46] Alizzi R.A., Xu D., Tenenbaum C.M., Wang W., Gavis E.R. (2020). The ELAV/Hu protein Found in neurons regulates cytoskeletal and ECM adhesion inputs for space-filling dendrite growth. PLoS Genet..

[47] Igaki T. (2009). Correcting developmental errors by apoptosis: Lessons from *Drosophila* JNK signaling. Apoptosis.

[48] Alfa R.W., Kim S.K. (2016). Using *Drosophila* to discover mechanisms underlying type 2 diabetes. Dis. Model. Mech..

[49] Chi C., Wang L., Lan W., Zhao L., Su Y. (2018). PpV, acting via the JNK pathway, represses apoptosis during normal development of *Drosophila* wing. Apoptosis.

[50] Igaki T., Kanda H., Yamamoto-Goto Y., Kanuka H., Kuranaga E., Aigaki T., Miura M. (2002). Eiger, a TNF superfamily ligand that triggers the *Drosophila* JNK pathway. EMBO J..

[51] Rajan A., Perrimon N. (2012). *Drosophila* cytokine unpaired 2 regulates physiological homeostasis by remotely controlling insulin secretion. Cell.

[52] Huang D.W., Sherman B.T., Lempicki R.A. (2009). Bioinformatics enrichment tools: Paths toward the comprehensive functional analysis of large gene lists. Nucleic Acids Res..

[53] Huang D.W., Sherman B.T., Lempicki R.A. (2009). Systematic and integrative analysis of large gene lists using DAVID bioinformatics resources. Nat. Protoc..

