# Supplementary material for: MicroRNA miR-274-5p Suppresses Found-in-Neurons Associated with Melanotic Mass Formation and Developmental Growth in Drosophila
Source: Insects. 2023 Aug 14;14(8):709. doi: 10.3390/insects14080709 (PMC10456003; doi:10.3390/insects14080709)
Supplement: Supplementary file 1 [file insects-14-00709-s001.zip › Supplementary FigureS1.pdf]

Supplementary Figure S1

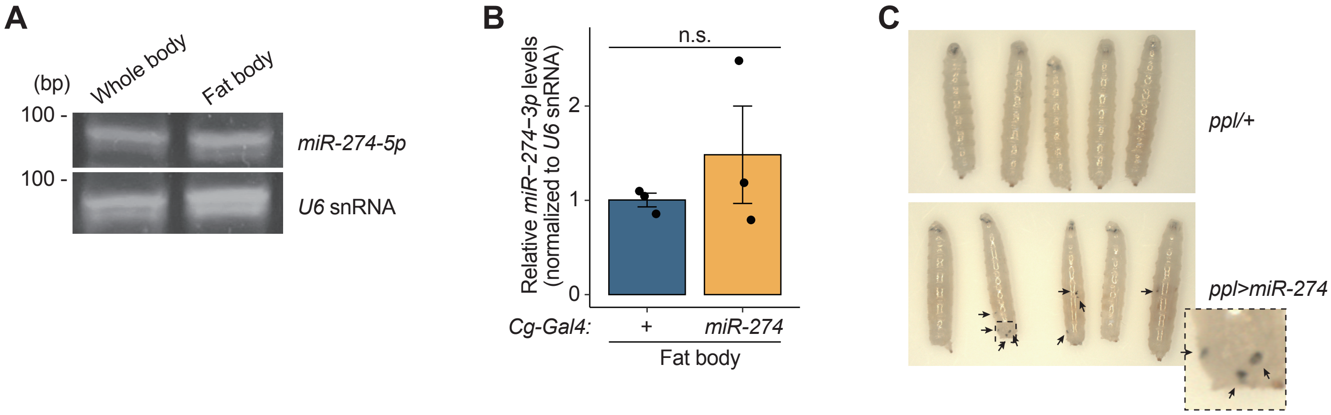

Supplementary Figure S1. Expression of *miR-274,* and the formation of melanotic masses in *ppl>miR-274* larvae. (**A**) Endogenous expression of *miR-274-5p* in the larval fat body. The expression levels of *miR-274-5p* were determined by semi-RT-qPCR in the whole body and the fat body of wandering third-instar larvae. *U6* snRNA served as an internal control. (**B**) Expression of *miR-274-3p* in the larval fat body of *Cg>miR-274*. Bar plots are shown as the mean ± SEM. n.s, not significant, as assessed by Student's t-test. (**C**) Wandering third-instar larvae of the indicated genotypes exhibiting melanotic masses. Melanotic masses are marked as arrows. The dashed box image is magnified.
