# Supplementary material for: MicroRNA miR-274-5p Suppresses Found-in-Neurons Associated with Melanotic Mass Formation and Developmental Growth in Drosophila
Source: Insects. 2023 Aug 14;14(8):709. doi: 10.3390/insects14080709 (PMC10456003; doi:10.3390/insects14080709)
Supplement: Supplementary file 1 [file insects-14-00709-s001.zip › Supplementary FigureS2.pdf]

Supplementary Figure S2

Figure 1G.

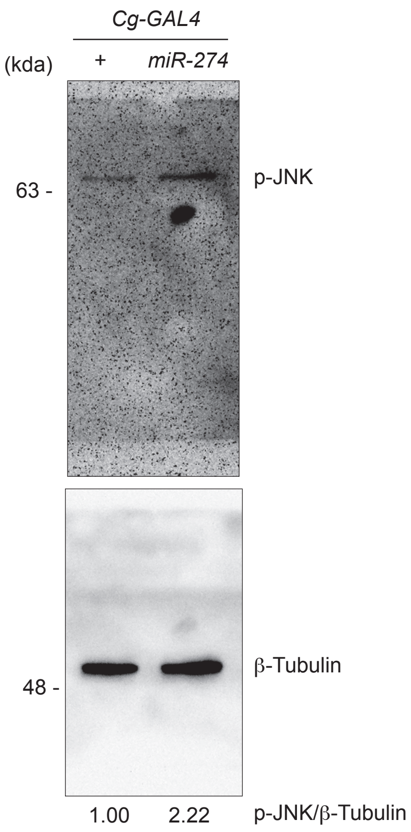

Figure 3A.

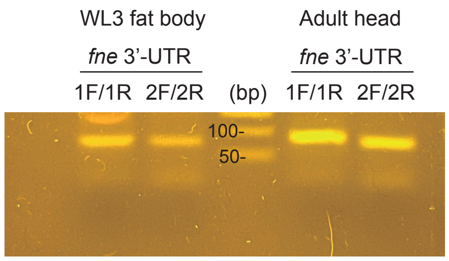

Figure 3B.

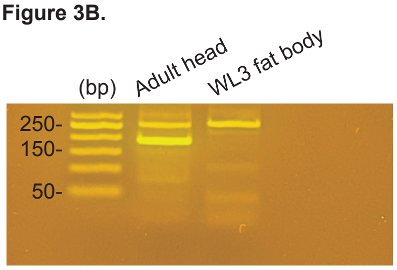

Figure 4G.

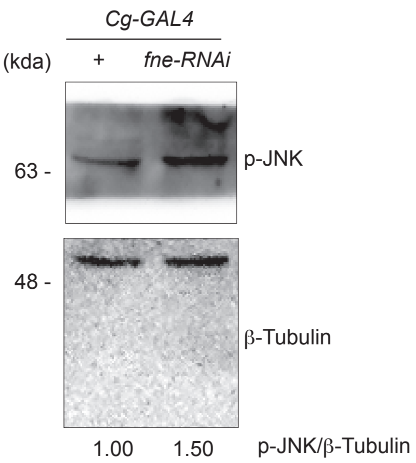

Supplementary Figure S2. Images of western blotting and DNA gel presented in the main figures.
